# The use of historical priors to improve the efficiency of phase II clinical trials with time-to-event endpoints

**DOI:** 10.1186/1745-6215-14-S1-P53

**Published:** 2013-11-29

**Authors:** Richard Jackson, Trevor Cox, Catrin Tudur-Smith

**Affiliations:** 1 University of Liverpool, Liverpool, UK

## 

We explore the possibility of incorporating historical information, which may take the form of data from previous trials, summary information or information derived from expert opinion into the design of phase II clinical trials with a time-to-event endpoint.

We model the survival data by means of the Piecewise Exponential Model and introduce a method by which a prior baseline hazard function is derived from summary information given by survival probabilities at given time points. We explore the use of gamma priors for the baseline hazard function as well as locally flat priors, where prior opinion of parameter estimates are equal within a given set of bounds.

We argue that placing priors on such information can improve decision making process by increasing the precision of the main parameter of interest, the hazard ratio. We further show that the influence of such a prior increases as more data are attributed to the experimental arm of a trial and argue that a prior on the baseline hazard function to be more appropriate than priors placed on the hazard ratio as there is typically much more information available on this aspect of a trial.

We illustrate the methodology via a simulation study to show how such an approach can improve measures such as the alpha level and power of comparisons between two therapies and make some arguments toward the case for unequal allocations. Applications to the ViP trial currently being run at the Liverpool Cancer Trials Unit are also discussed.

